# Building an ethical environment improves patient privacy and satisfaction in the crowded emergency department: a quasi-experimental study

**DOI:** 10.1186/1472-6939-14-8

**Published:** 2013-02-20

**Authors:** Yen-Ko Lin, Wei-Che Lee, Liang-Chi Kuo, Yuan-Chia Cheng, Chia-Ju Lin, Hsing-Lin Lin, Chao-Wen Chen, Tsung-Ying Lin

**Affiliations:** 1 Department of Emergency Medicine, Kaohsiung Medical University Hospital, Kaohsiung Medical University, Kaohsiung, Taiwan; 2 Division of Traumatology, Department of Surgery, Kaohsiung Medical University Hospital, Kaohsiung Medical University, Kaohsiung, Taiwan; 3 College of Nursing, Kaohsiung Medical University, Kaohsiung, Taiwan

**Keywords:** Ethical environment, Privacy, Confidentiality, Satisfaction, Emergency Department

## Abstract

**Background:**

To evaluate the effectiveness of a multifaceted intervention in improving emergency department (ED) patient privacy and satisfaction in the crowded ED setting.

**Methods:**

A pre- and post-intervention study was conducted. A multifaceted intervention was implemented in a university-affiliated hospital ED. The intervention developed strategies to improve ED patient privacy and satisfaction, including redesigning the ED environment, process management, access control, and staff education and training, and encouraging ethics consultation. The effectiveness of the intervention was evaluated using patient surveys. Eligibility data were collected after the intervention and compared to data collected before the intervention. Differences in patient satisfaction and patient perception of privacy were adjusted for predefined covariates using multivariable ordinal logistic regression.

**Results:**

Structured questionnaires were collected with 313 ED patients before the intervention and 341 ED patients after the intervention. There were no important covariate differences, except for treatment area, between the two groups. Significant improvements were observed in patient perception of “personal information overheard by others”, being “seen by irrelevant persons”, having “unintentionally heard inappropriate conversations from healthcare providers”, and experiencing “providers’ respect for my privacy”. There was significant improvement in patient overall perception of privacy and satisfaction. There were statistically significant correlations between the intervention and patient overall perception of privacy and satisfaction on multivariable analysis.

**Conclusions:**

Significant improvements were achieved with an intervention. Patients perceived significantly more privacy and satisfaction in ED care after the intervention. We believe that these improvements were the result of major philosophical, administrative, and operational changes aimed at respecting both patient privacy and satisfaction.

## Background

Respect for patient privacy and assuring patient confidentiality have been regarded as essential obligations of healthcare providers and primary responsibilities of healthcare institutions [1-3]. Emergency departments (EDs) in hospitals, unlike other units, do not have private and semiprivate rooms to help protect privacy and confidentiality. EDs usually have treatment bays; most are separated only by curtains, and patients are placed close to one another for long periods of time. Several studies have reported frequent infringements of privacy and confidentiality in hospital EDs [1,4]. In the ED, lack of privacy and confidentiality make communication difficult between patients and healthcare providers, especially when they discuss sensitive medical conditions and some important treatment options. It may result in misdiagnosis or medical errors for healthcare providers and ineffective treatments for patients. As such, it may erode the patient’s trust and make it difficult to build a good patient-physician relationship.

In particular, ED crowding has become a common and significant issue [5-7]. The ED physical and environmental limitations and high volume of patients have made the protection of patient privacy and confidentiality even more difficult. When the ED becomes crowded, patients have to be placed in or near hallways, greatly exacerbating the challenges of protecting privacy and ensuring confidentiality [5,8,9]. Therefore, it is the top priority for patient-centered health care to build a renovated ED with respect for ED patient privacy and confidentiality.

The ethical environment in a healthcare organization may be conceptualized by how the organization influences the ability of healthcare providers to advance what they ought to do in their daily work [10]. Research has shown that an ethical environment and climate have a positive effect on healthcare providers’ daily work in terms of job satisfaction, retention at work, ability to manage conflict, and so on [10-14], as well as patient outcome [10]. The leaders of the organization play a key role in building, maintaining, and encouraging an ethical environment and climate. It is the main responsibility of the leadership in the organization to create and sustain an ethical environment and climate, because these not only stand for what ought to be done but also encourage good practice [15]. Therefore, the leaders should devote all their efforts to fostering an ethical environment and climate in the organization [16].

This study aimed to examine the effect of an intervention targeting ED patient privacy and confidentiality as well as satisfaction. Multivariable analyses controlled for the confounding effects of predefined covariates. The investigators tried to determine if the intervention would improve patient privacy and satisfaction.

## Methods

We conducted a quasi-experimental study. Patient responses before and after the intervention were analyzed to determine if there was a difference in patient perception of privacy and satisfaction. A prospective convenience sample of ED patients was surveyed. Eligibility data were collected from September 13, 2010, to October 1, 2010 for the post-intervention group and compared to our previous survey collected from July 7, 2008, to July 25, 2008 for the pre-intervention group. Patients were approached during weekdays between the hours of 9 a.m. and 4 p.m.

The study was conducted at an urban academic ED in the city of Kaohsiung in Southern Taiwan, with an emergency medicine residency and an annual census of 84,000 patients in 2008, which had increased to approximately 89,000 in 2010. Most physicians and nurses who cared for patients during the pre- and post-intervention periods of the study were the same personnel (given a low staff turnover rate plus some new staff in our department). The study was approved by the Institutional Review Board of Kaohsiung Medical University Hospital.

All patients discharged from the ED were eligible. Exclusion criteria included medical incapacitation, inability to speak or read Mandarin, refusal to participate, and age younger than 18 years. Patient consent to participate in the study and complete a self-administered questionnaire regarding their ED stay from the perspectives of their privacy and satisfaction was requested. A one-page questionnaire was developed and administered to all eligible patients. Patient responses were kept confidential. Collected data included age, gender, and treatment area in the ED (registered bed or hallway). The length of stay for each patient was recorded from the ED computer system. These variables were used in subsequent multivariable analyses. The questionnaire and the inclusion/exclusion criteria were the same before and after the intervention.

The questionnaire was developed to assess the patient’s perception of privacy and satisfaction with emergency care based on the conceptual framework proposed by the literature [1,17-20]. Four forms of privacy that limited access to the person including physical privacy, informational privacy, decisional privacy, and proprietary privacy were proposed [17]. The internal consistency of these questions assessing measures of privacy for emergency care was evaluated, and a high consistency for these ratings was revealed [21].

We developed the intervention using the conceptual model proposed by McDaniel et al. [10], in which an ethical environment contributed to providers’ work and patient outcomes. Building an ethical environment has been the ultimate goal in our hospital and we believe that this should be developed through various targets, which may enable evaluation of the effectiveness of the intervention. For logistical reasons, the ED was chosen as the first priority target department.

Under the leadership of the director and patient care manager, a multidisciplinary quality improvement team was formed to improve the quality of patient care in our ED. The team included one senior hospital administrator (deputy superintendent), appropriate physicians, a nursing leader, and ancillary personnel. The ethicist, who helped identify the ethical issues and who responds to ethical consultations and concerns, was also included on the team. The team members met regularly to discuss, observe, collect and analyze relevant data, then formed and presented recommendations to the senior hospital administrator.

The team members evaluating the ED environment found numerous challenges. The most important ethical issue identified was the ED environment concerning patient privacy and safety as well as satisfaction. This issue had been discussed by team members, approved by the senior hospital administrator, and considered as the top priority that should be managed for our ED care. The theoretical construct centered on the belief that environmental, operational, and process changes along with staff education were required to improve patient privacy and satisfaction.

Our ED contains only curtained rooms. Most patients spend most of their time in one room for their ED stay. Rooms are randomly assigned to patients based upon availability. Patients are placed in the hallways when no room is available. Rooms are frequently full in our ED, and many patients have to be placed in the hallway for a longer period of time during their stay in the ED.

Before the implementation of the intervention, the ED had been openly accessible to the public without access control. There is only one exterior entrance to the ED in our hospital; it is a public entrance supervised by 24-hour guard security. Ambulances may offload patients they have transported, and walk-in patients may enter the triage and waiting area. In our ED, there are several cameras to audit the public area, such as hallways, although they are not used to audit the treatment area to protect patient privacy and confidentiality. The ED is, in fact, always a high risk area with potential threats to safety for patients and healthcare providers. In the past few years, workplace violence has occurred in our ED to our healthcare providers by patients and families. Moreover, a lot of visitors and staff usually use the ED as a pathway to travel in and out of the hospital or from one part of the hospital to another. These uses might result in jeopardizing patient privacy and safety. Therefore, the ED, as a high risk area, needed another layer of safety protection and control.

The team identified a lack of vigilance during the process of patient care as another constraint in protecting patient privacy, to the extent that it may result in patients withholding personal information and having reluctance to be physically examined. Ideally, healthcare providers should not discuss patient information in open areas, such as hallways, where it may be overheard by irrelevant persons. In the ED, healthcare providers usually discuss patient conditions in the treatment bays or workstations. Sometimes, healthcare providers have neglected to use private treatment rooms or movable screens to separate patients when examinations or procedures have had to be performed on patients in open areas. Therefore, it is essential to continue the education of healthcare providers to be sensitive to and avoid infringement on patient privacy as much as possible [21,22].

Accordingly, team members planned to conduct an intervention to improve patient privacy and satisfaction, including ED environmental space reorganization, streamlined redesign, process management, access control, staff education and training, and encouragement of ethics consultation. The intervention was discussed and initiated with the support of senior hospital administrators and the ED quality assurance committee. The intervention is described in Table 1. All of the changes were achieved gradually over the two years from 2008 to 2010.

**Table 1:** The study intervention

**Intervention**	**Description**
Environmental space reorganization and process management	We reorganized space and assigned space utility according to different treatment purposes. An observation area was set aside for those patients expecting to stay in the ED for a longer period of time (usually more than 6 hours) for observation or later admittance to the ward. The observation area was set up in the inner area of the ED with a quieter and more private environment equipped with healthcare providers caring for these patients. Patients would be moved from the treatment area to the observation area when necessary. However, patients would be moved back to the treatment area if medical conditions warranted it.
Access control	Limited access to the treatment area would protect patient privacy and safety as well as allow healthcare providers to function without interference. To avoid the ED being open to the public and used as an access way, a card reader system was introduced to allow the ED staff, delivery personnel, and other authorized persons to access the ED. An entry sign was placed to explain why the access control was essential for assisting in the protection of patient privacy and safety.
Bioethics education and training	**Institutional level**
	Many educational activities for continuing further staff education and training after the implementation of the intervention were introduced. A one-day workshop per month was provided during August to October in 2008 for all hospital healthcare providers and dealt with clinical ethical issues in terms of ethics consultation, privacy and confidentiality, disclosure of medical information, and professionalism, etc. Our management concept, emphasizing adherence to medical ethics and accountability for social responsibility, were reaffirmed and all hospital healthcare providers were educated about this. Moreover, special speech training for patient privacy is held annually for all healthcare providers, as well as educational training for all new medical students, residents, and staff.
	**ED level**
	In collaboration with the education of all hospital healthcare providers, we coordinated case conference meetings at the ED monthly, shaping policies and implementing strategies for improving ED patient care in terms of privacy, confidentiality and satisfaction. The ED staff was trained to be sensitive to patient privacy and ensure confidentiality. For example, performing procedures and physical examinations in the public areas should be avoided if at all possible. Healthcare providers were reminded to use the treatment rooms or movable screens to protect patient privacy, to be aware of the surrounding environment concerning patient information being overheard by others, to keep their voices down when discussing patient information, and to avoid leaving notes with patient information and charts in the public areas that could be easily accessed by irrelevant persons, etc.
	Feedback from healthcare providers was collected after each educational activity, and most staff responded with satisfaction and acknowledged that the education and training would be helpful for their practice and patient care.
Encouraging ethics consultation	The staff have encouraged ethics consultation to allow patients to express their ethical concerns. Some seed staff members were selected and specially educated to provide peer support for other staff regarding ethical concerns in their own departments.

The primary outcome was patient perception of overall privacy and satisfaction measured on an ordinal 5-point Likert scale. Secondary measures concerned patient privacy and confidentiality, including “personal information overheard by others”, being “seen by irrelevant persons”, having “unintentionally heard inappropriate conversations from healthcare providers”, and experiencing “providers’ respect for my privacy” measured on an ordinal 5-point Likert scale. All outcomes were patient-reported.

Baseline characteristics of patients for the pre-intervention and post-intervention groups were compared. The Fisher exact test was conducted for binary, ordinal, and categorical variables. The Wilcoxon rank sum test was conducted for continuous and ordinal variables. A multivariable ordinal logistic regression model of patient privacy and satisfaction as well as secondary outcomes measured on a 5-point Likert scale with predefined covariates were performed, and likelihood ratio tests for the multivariable models were performed. All data analysis was performed with the Stata version 10.0 (StataCorp, College Station, TX).

## Results

Three hundred and sixty-four patients were approached to complete the questionnaire, and 313 patients (86%) did so in the pre-intervention group. In the post-intervention group, 386 patients were approached to complete the questionnaire and 341 patients (88%) did so. Baseline characteristics for pre and post-intervention patients are presented in Table 2. There were no important differences for age, gender, and length of stay in the ED before and after the intervention. There was a statistical difference for the treatment area between pre- and post-intervention groups. Patients in the post-intervention group had a higher rate of being treated in the hallway.

**Table 2:** Baseline characteristics

**Characteristic**	**Pre-intervention**	**Post-intervention**	**p-value**
	**(n=313)**	**(n=341)**	
Age, y, No (%)			0.12
<20	13 (4.2)	30 (8.8)	
20–29	43 (13.7)	53 (15.5)	
30–39	65 (20.8)	71 (20.8)	
40–49	79 (25.2)	71 (20.8)	
50–59	63 (20.1)	61 (17.9)	
60–69	22 (7.0)	33 (9.7)	
>69	28 (9.0)	22 (6.5)	
Males, No (%)	141 (45.1)	169 (49.6)	0.25
Treatment area			0.003
Registered bed	231 (73.8)	215 (63.1)	
Hallway	82 (26.2)	126 (36.9)	
Length of stay, min	Median, 147	Median, 140	0.38

Primary outcomes measured on a 5-point scale are listed in Table 3. There were statistically significant differences between pre- and post-intervention groups on “Overall perception of privacy” and “Satisfaction”. Secondary outcomes measured on a 5-point scale are listed in Table 4. There were statistically significant differences between pre- and post-intervention groups on “Personal information overheard by others”, “Seen by irrelevant persons”, “Unintentionally heard inappropriate conversations from healthcare providers”, and “Providers’ respect for my privacy”. However, there was no statistically significant difference between pre- and post-intervention groups on “Space provided privacy when physically examined”.

**Table 3:** Comparison of overall perception of privacy and satisfaction between pre-intervention and post-intervention groups

**Outcome**	**Pre-intervention**	**Post-intervention**	**p-value**
	**No (%)**	**No (%)**	
Overall perception of privacy			0.000
Very good	32 (10.2)	44 (12.9)	
Good	126 (40.3)	200 (58.7)	
Fair	132 (42.2)	80 (23.5)	
Poor	15 (4.8)	15 (4.4)	
Very poor	8 (2.6)	2 (0.6)	
Satisfaction			0.000
Very good	28 (9.0)	83 (24.3)	
Good	129 (41.2)	181 (53.1)	
Fair	127 (40.6)	66 (19.4)	
Poor	23 (7.4)	10 (2.9)	
Very poor	6 (1.9)	1 (0.3)	

**Table 4:** Comparison of secondary outcomes between pre-intervention and post-intervention groups

**Outcome**	**Pre-intervention**	**Post-intervention**	**p-value**
	**No (%)**	**No (%)**	
Personal information overheard by others			0.000
Strongly agree	9 (2.9)	10 (2.9)	
Agree	64 (20.5)	56 (16.4)	
Fair	137 (43.8)	96 (28.2)	
Disagree	78 (24.9)	71 (20.8)	
Strongly disagree	25 (8.0)	108 (31.7)	
Seen by irrelevant persons			0.000
Strongly agree	10 (3.2)	12 (3.5)	
Agree	48 (15.3)	27 (7.9)	
Fair	97 (31.0)	34 (10.0)	
Disagree	106 (33.9)	100 (29.3)	
Strongly disagree	52 (16.6)	168 (49.3)	
Unintentionally heard inappropriate conversations from healthcare providers			0.000
Strongly agree	11 (3.5)	1 (0.3)	
Agree	23 (7.4)	5 (1.5)	
Fair	91 (29.1)	68 (19.9)	
Disagree	120 (38.3)	86 (25.2)	
Strongly disagree	68 (21.7)	181 (53.1)	
Space provided privacy when physically examined			0.97
Strongly agree	46 (14.7)	45 (13.2)	
Agree	129 (41.21)	155 (45.5)	
Fair	105 (33.6)	94 (27.6)	
Disagree	27 (8.6)	40 (11.7)	
Strongly disagree	6 (1.9)	7 (2.1)	
Providers’ respect for my privacy			0.000
Very good	47 (15.0)	95 (27.9)	
Good	155 (49.5)	193 (56.6)	
Fair	93 (29.7)	47 (13.8)	
Poor	16 (5.1)	5 (1.5)	
Very poor	2 (0.6)	1 (0.3)	

Multivariable ordinal logistic regression models of primary outcomes controlling for predefined covariates are presented in Table 5. The adjusted odds ratio for the post-intervention group suggests that the intervention improved patient overall perception of privacy and satisfaction. Adjusted odds ratio for overall perception of privacy and satisfaction in this group was 2.367 (95% confidence interval 1.740 to 3.221) and 4.004 (95% confidence interval 2.921 to 5.488), respectively. Similar analyses for “Personal information overheard by others”, “Seen by irrelevant persons”, “Unintentionally heard inappropriate conversations from healthcare providers”, and “Providers’ respect for my privacy” demonstrated important improvements for the post-intervention group (Table 6).

**Table 5:** Multivariable ordinal logistic regression model for overall perception of privacy and satisfaction

	**Overall perception of privacy**		**Satisfaction**	
	**Odds ratio**	**95% CI**	**Odds ratio**	**95% CI**
**Post-intervention group**				
(reference group=Pre-intervention group)	2.367**	1.740–3.221	4.004**	2.921–5.488
**Age, y**	0.996	0.987–1.005	1.006	0.997–1.015
**Treatment area**				
(reference group=registered bed)	0.354**	0.256–0.490	0.367**	0.266–0.505
**Length of stay, min**	0.997**	0.996–0.998	0.998**	0.997–0.999
Likelihood ratio test for model	*χ2*= 89.30; *P* < 0.000		*χ2*= 115.8; *P* < 0.000	

*p< 0.05; **p<0.01. Sample size of regression model = 654.

**Table 6:** Multivariable ordinal logistic regression model for secondary outcomes

	**Personal information overheard by others**		**Seen by irrelevant persons**		**Unintentionally heard inappropriate conversations from healthcare providers**		**Space provided privacy when physically examined**		**Providers’ respect for my privacy**	
	**Odds ratio**	**95% CI**	**Odds ratio**	**95% CI**	**Odds ratio**	**95% CI**	**Odds ratio**	**95% CI**	**Odds ratio**	**95% CI**
**Post-intervention group**										
(reference group= Pre-intervention group)	0.462**	0.347–0.615	0.246**	0.182–0.333	0.306**	0.227–0.412	1.078	0.810–1.436	2.852**	2.091–3.889
**Age, y**	0.991*	0.982–0.999	0.982**	0.973–0.991	0.997	0.988–1.006	1.008	0.998–1.017	1.013**	1.004–1.022
**Treatment area**										
(reference group=registered bed)	0.742	0.550–1.003	1.103	0.810–1.501	0.888	0.650–1.213	0.564**	0.415–0.767	0.618**	0.450–0.851
**Length of stay, min**	1.000	0.999–1.002	1.002**	1.000–1.002	1.000	0.999–1.001	0.999	0.998–1.000	0.999	0.998–1.000
Likelihood ratio test for model	*χ2*= 39.04; *P* < 0.000		*χ2*= 107.39; *P* < 0.000		*χ2*=67.49; *P* < 0.000		*χ2*=17.87; *P* < 0.001		*χ2*= 55.10; *P* < 0.000	

*p< 0.05; **p<0.01. Sample size of regression model = 654.

## Discussion

We report the result of an intervention to improve patient privacy and satisfaction in a university-based ED. An important association between outcomes and intervention on bivariate analysis has been identified, and was established as significant after controlling for multiple, predefined confounders in multivariable analyses. Patient perception of privacy and satisfaction improved after the implementation of the intervention, as did patient perception of healthcare providers’ respect for their privacy.

As an institutional priority, and with full integration of all the departments and the emergency medicine staff in the intervention, major improvements were noted. We believe that several factors were responsible for major improvements from the intervention. These include: (1) emphasis on ED environmental space reorganization, (2) process management and streamlined redesign, (3) access control, (4) staff bioethics education and training, and (5) encouraging ethics consultation.

Patient privacy in the ED is easily compromised by physical design, crowding, or lack of healthcare provider vigilance [2,4,5]. Sohrabia and Alimohammadib reported that the target teaching hospitals in their survey did not provide appropriate privacy, confidentiality or enough facilities, all of which should be considered in designing teaching hospitals for the patients and the staff. By redesigning the environment, patient satisfaction and staff job satisfaction might improve [23]. Lovato et al. reported that interventions, including introduction of a new triage “silver code”, review of criteria for pediatric triage, creating a new triage room with a dedicated nurse, improvement of the waiting room, creation of a waiting room specifically for pediatric patients, and introduction of volunteers to humanize the ED, may improve patient satisfaction [24]. Moreover, continuing education and training in bioethics are essential for healthcare providers to be sensitive to ethical issues and provide good practice. In some studies, the authors emphasized that education and formal training in bioethics are important to make that sure clinical practice adheres to principles of ethics to promote a high quality of care [4,25]. Furthermore, some authors emphasize that facilitating ethics education and ethics consultation in the institution will help develop the ethics capacity and resources to assist healthcare providers, and in this way demonstrate the organization’s commitment to establish a positive ethical climate and environment [16].

Our results confirmed significant improvements in patient perceptions of “personal information overheard by others”, being “seen by irrelevant persons”, having “unintentionally heard inappropriate conversations from healthcare providers”, and experiencing “providers’ respect for my privacy”. There was significant improvement in overall patient perception of privacy and satisfaction. There were statistically significant correlations between the intervention and overall patient perception of privacy and satisfaction on multivariable analysis. We believe that the ED environmental and process redesign resulted in a decrease of patients overhearing or being overheard (concerning inappropriate information) and being seen by irrelevant persons. Rooms close to the staff work area have the most opportunity for breaching patient privacy and confidentiality [4]. In one study, the investigators reported that both an increase in the department size and the elimination of rooms separated only by curtains might significantly reduce patients overhearing conversations about themselves or other patients [8]. We redesigned our ED space and process to provide an inner and private space away from the workstation for patients under observation or waiting for admittance. We believe this change led to improved patient privacy. Access control led to significant decreases in visitors and bystanders as well as staff transiting via the ED. This should greatly improve patient safety and patient perceptions of privacy. Another major improvement, we believe, was the change of the attitude and behavior of healthcare providers in being more sensitive to and respectful of patient privacy and confidentiality, via education and training programs and the encouragement of ethics consultation. Some studies have emphasized the importance of staff education in improving patient privacy and satisfaction [21,26]. We could improve patient perceptions of privacy by using a lower voice tone when discussing treatment options with patients, and by avoiding discussions of patient information in treatment areas or open workstations where they could easily be overheard by anyone nearby. Accordingly, we believe that these improvements were the result of major administrative, philosophical, and operational changes aimed at respecting both patient privacy and satisfaction.

Moreover, the importance of the commitment of senior hospital administrators and the multidisciplinary quality improvement team should be emphasized. These major improvements could not have been achieved without strong commitment and support by senior hospital administrators and the proper application of organizational resources by using a multidisciplinary improvement team approach.

There was no significant improvement in patient perceptions that the “space provided privacy when physically examined” in our study. This might be due to the physical limitations of our ED. Our ED has only curtained rooms with smaller spaces for each room. Some studies have reported that ED patients might perceive less auditory and visual privacy when staying in curtained treatment areas than in rooms with solid walls [4,18,19]. Private rooms with solid walls should be provided when patients are undergoing physical examinations, especially when private body parts are examined. Ultimately, taking this into consideration would provide a more permanent solution to the problem in the reconstruction and renovation of EDs.

ED crowding has been a significant and increasing longstanding issue [5-7,27-29]. When the ED has become crowded, patients have had to be placed in proximity to one another in the treatment areas or in hallways for long periods, and this may exacerbate the problem of protecting patient privacy and confidentiality. In one study, the results revealed that patients treated in hallways were significantly more unsatisfied than those treated in the regular registered bed areas [21]. ED physical and environmental limitations and high volumes of patients have made the protection of patient privacy and confidentiality more difficult. Many strategies have been considered to overcome the problem of ED crowding [6,27,30-33]. When ED crowding is overcome, with overall patient volume reduced, it is possible to further decrease patient length of stay and minimize patient waiting time in the ED to improve patient privacy and confidentiality.

Though the intervention succeeded in improving ED patient privacy and satisfaction, it should be emphasized that major improvement was achieved by building an ethical environment for daily work activities. Though we did not compare changes in the ethical environment and climate between pre- and post-intervention, we believe that the improvement in patient outcomes has reflected these achievements. Institutions, on one hand, should emphasize patient-centered health care as a top priority, and on the other should build an ethical environment and climate to improve quality of care in the ED. ED staff must often share protected health information with other staff and healthcare personnel to provide appropriate care for the patient.

Our study had several limitations. First, because it was not a randomized controlled study design, there might be many confounders limiting our inferences, as noted by Eccles et al. in their discussion of research designs for evaluating the effectiveness of change and improvement strategies [34]. Because it is difficult to obtain comparable samples for the comparison of patient outcomes before and after organizational changes within an institution and given other logistical factors for this study, a multicenter randomized controlled study will be needed to confirm the results. Second, the response rates were different between pre- and post-intervention groups; this might reflect how aggressively we approached the patients, and might have had an influence on our results. However, there was no difference between the two groups in baseline characteristics in our study. Although treatment areas between the two groups were different with more patients being treated in the hallways in the post-intervention group, this should have worked against improvement in patient perceptions of privacy and satisfaction, which were nonetheless greater for the post-intervention group. Future studies are needed to confirm these results. Third, the survey time points were different. The numbers of the ED census and the diseases and medical problems of the patients in the two study periods might be different. These might have influenced our results. Moreover, the medical conditions of patients might have an influence on their perceptions of patient privacy and satisfaction. Future studies are needed to explore these associations. Finally, the study was conducted in one institution with discharged, ambulant patients, and the results might not be generalizable to other institutions and admitted patients.

## Conclusions

Significant improvements were achieved with an intervention of environmental space reorganization and process management, access control, staff education and training, and encouraging ethics consultation. Patients perceived significant improvements in privacy and satisfaction for their ED care after the intervention. We believe that these improvements were the result of major philosophical, administrative, and operational changes aimed at respecting both patient privacy and satisfaction. Institutions should give top priority to patient-centered health care, and build an ethical environment to improve quality of care.

## Competing interests

The authors declare that they have no competing interests.

## Authors’ contributions

YKL and CJL carried out the study conception and design. TYL and HLL carried out the acquisition of data. LCK and WCL performed the analysis and interpretation of data. YKL and CJL carried out the drafting the manuscript. YCC and CWC participated in the critical revision. All authors read and approved the final manuscript.

## Pre-publication history

The pre-publication history for this paper can be accessed here:

http://www.biomedcentral.com/1472-6939/14/8/prepub
